# Merton’s problem for an investor with a benchmark in a Barndorff-Nielsen and Shephard market

**DOI:** 10.1186/s40064-015-0842-9

**Published:** 2015-02-24

**Authors:** Jan Lennartsson, Carl Lindberg

**Affiliations:** Department of mathematical sciences, Chalmers university and Gothenburg university, Chalmers tvärgata 4, Göteborg, Sweden; Swedish Ap-fund and Uppsala university, Östra hamngatan 26-28, Box 11155, Göteborg, 404 24 Sweden

**Keywords:** Stochastic control, Portfolio optimization, Stochastic volatility, Benchmark, HJB equation

## Abstract

To try to outperform an externally given benchmark with known weights is the most common equity mandate in the financial industry. For quantitative investors, this task is predominantly approached by optimizing their portfolios consecutively over short time horizons with one-period models. We seek in this paper to provide a theoretical justification to this practice when the underlying market is of Barndorff-Nielsen and Shephard type. This is done by verifying that an investor who seeks to maximize her expected terminal exponential utility of wealth in excess of her benchmark will in fact use an optimal portfolio equivalent to the one-period Markowitz mean-variance problem in continuum under the corresponding Black-Scholes market. Further, we can represent the solution to the optimization problem as in Feynman-Kac form. Hence, the problem, and its solution, is analogous to Merton’s classical portfolio problem, with the main difference that Merton maximizes expected utility of terminal wealth, not wealth in excess of a benchmark.

## Introduction

Classical portfolio optimization literature focus on the problem of maximizing expected utility of wealth at a deterministic future time. While this may be an intuitive problem setting, to measure performance in absolute terms, the dominating approach in the financial industry is rather to measure performance in excess of an externally given benchmark index. The return in excess of the benchmark is called *alpha*. Measuring performance in this manner better captures the skill of the individual investor.

Portfolio optimization started with Markowitz’ landmark paper, see (Markowitz 1952), where the investors’ conflicting objects of high return versus low risk was quantified. Since then, the field has been generalized and refined and sophisticated stochastic control models have been proposed for making optimal investment decisions. In continuous time, Merton solved the problem of optimal allocation of a portfolio in order to maximize the expected utility of wealth in a Black-Scholes market in (Merton 1969). For optimality with state-dependent risk aversion see (Björk et al. 2014). To incorporate more features from actual financial markets different stochastic volatility models have been suggested. For portfolio optimality in state-dependent Markov markets see (Celikyurt and Özekici 2007). For the Heston model see (Kraft 2005). Barndorf-Nielsen and Shephard (B-NS) introduced in (Barndorff-Nielsen and Shephard 2001) a stochastic volatility model where the stock prices are non-homogeneous geometric Brownian motions, and the dynamics of the multidimensional appreciation rate and volatility matrix are driven by non-Gaussian Ornstein-Uhlenbeck processes. A nice property of this model is that it allows for very sudden increases in volatility, which is a phenomenon often encountered in financial markets. For a single stock B-NS market, Benth solved the portfolio allocating problem in (Benth et al. 2003). For a multiple stock market the problem was addressed and solved in (Lindberg 2006a) and (Lindberg 2006b). Kallsen and Muhle-Karbey solved a similar problem using a martingale approach in (Kallsen and Muhle-Karbey 2010). Point estimation in the B-NS market is done in (Lindberg 2007).

A single investor hardly dictates the performance of the market and absolute wealth depends directly on the overall market fluctuations. By investigating the relative wealth, defined as the difference of wealth between the portfolio and the benchmark index, we have an isolated measure of the investor’s performance which does not depend on the underlying market. In order for the relative wealth to be independent of the benchmark, it is necessary that the benchmark is continously rebalanced, see (Korn and Lindberg 2013). The level of wealth of the benchmark is set to be equal to that of the personal investor. Mathematically, the rebalancing of the benchmark is being done continuously. However, in practice it is often done once a day or even more seldom. The adaptation of the benchmark wealth is made to avoid that the investor’s performance will be affected by the so called *beta effect*. The term beta effect is used to describe the situation when the performance of the investor, which is measured only in terms of its performance in excess of the benchmark, start to be affected notably on the *absolute* performance of the benchmark. This happens if the investor’s capital is considerably larger, or smaller, than the capital held in the benchmark. This implies that we are evaluating performance in excess of a non self-financing portfolio. It sums over the difference between investor’s daily profit minus the daily profit of the current benchmark. Hence, the concept of relative wealth is merely an abstraction, but nevertheless an industry ubiquity.

The problem of maximizing utility of wealth in excess of a benchmark in a standard Black-Scholes market was recently solved in (Korn and Lindberg 2013). We consider the corresponding problem in the B-NS market.

The financial industry standard, regarding how portfolio optimization is applied in practice, is to rebalance one-period mean variance portfolios over short consecutive time horizons. It is been unknown to what extent this "local" optimization approach actually yields good results also in the long run. We will show here that the optimal portfolio in terms of exponential utility of relative wealth in a B-NS market replicates the optimal portfolio of the corresponding Markowitz mean-variance problem in continuum. That is, by continuously rebalancing one-period benchmark relative mean-variance solutions one replicates the optimal portfolio for an investor maximizing expected utility of terminal wealth. This is actually completely analogous to (Merton 1969), and the differences between his paper and the present one are small (set aside that we use a stochastic volatility model). Mainly, the difference is that Merton aims at finding an optimal portfolio on an absolute level while we consider an optimal *alpha* portfolio. Merton considers strategies as being fractions of wealth and his optimal strategy - the local mean-variance strategy the investor should apply continuously - has the constraint that the sum of all portfolio weights should be equal to one. We, in the other hand, view the strategies in terms of capital, and use the constraint that the sum of all portfolio weights should sum to zero, i.e. that the net exposure relative to the benchmark should be zero. With Merton’s problem, the optimal strategy amounts to solving the stochastic control problem of maximizing expected utility of terminal wealth. Analogously, for our problem the optimal strategy amounts to maximizing expected utility of terminal wealth *in excess of the benchmark*.

It is natural to ask whether we have almost sure nonnegativity of the investor’s total wealth for the portfolio problem we consider. The answer to this quesion is affirmative, since we can choose the bounds on our portfolio weights such that the investor’s total portfolio holdings remain positive. In practice, this constraint is often active for stocks which have small index weights.

We solve the present portfolio problem using the corresponding Hamilton-Jacobi-Bellman (HJB) equation. The paper is structured in the following way. In Section 2 model parameters such as the market model, utility function and optimal value function are defined. We set up the stochastic control problem in Section 3, and we also reformulate the problem in terms of its associated HJB equation. In Section 4, we prove that the optimization problem is well defined. We prove in Section 5 a verification theorem. In Section 6, a well defined explicit solution to the HJB equation is given. The solution to the HJB equation is verified to be a solution to the optimization problem in Section 7.

## The model

In this section we set up the market model, including the governing dynamics and the investor’s utility function. Further, we introduce the relative portfolio - the investor’s portfolio holdings minus her benchmark portfolio - in order to set up the optimal value function.

### 2.1 The market model

Suppose that for 0≤*t*≤*T*<*∞* a complete probability space $(\Omega,\mathcal {F},P)$ is given with a corresponding filtration, $\left \{\mathcal {F}_{t}\right \}_{0\leq t\leq T}$, satisfying the usual conditions. In addition, $\left \{{Z_{t}^{i}}\right \}_{i=1,...,k}$ denotes *k* independent cádlág subordinators, and $\left \{{W_{t}^{i}}\right \}_{i=1,...,m}$ is a set of *m* independent Brownian motions independent of the subordinators. The filtration to be considered is

$$\mathcal{F}_{t}=\left\{\left\{{W_{s}^{i}}\right\}_{i=1,...,m},\left\{Z_{\lambda_{i}s}^{i} \right\}_{i=1,...,k}:s\leq t\right\}, $$

where $\lambda \in \mathbb {R}_{+}^{k}$. Subsequently,  in superscript will denote transpose. Boldface numbers denote column vectors of suitable size with each element equal to the boldfaced number. I.e. **1** denotes a vector of ones and **0** denotes a vector of zeros. Throughout the paper, the notation indicating conditional properties will be omitted when there is no risk of confusion.

The frictionless market is modelled as the multidimensional B-NS factor model defined in (Lindberg 2006b), and the market is equipped with *n*≤*m* stocks

$$S_{t}=\left({S_{t}^{1}},{S_{t}^{2}},...,{S_{t}^{n}}\right)^{\mathcal{T}} $$

and *k* news processes

$$Y_{t}=\left({Y_{t}^{1}},{Y_{t}^{2}},...,{Y_{t}^{k}}\right)^{\mathcal{T}}. $$

The dynamics of the system is defined by the stochastic differential equations

(1) $$\begin{array}{@{}rcl@{}} {dS}_{t} & =&S_{t}.\left(\mu_{t}dt+\sigma_{t}{dW}_{t}\right) \end{array} $$

(2) $$\begin{array}{@{}rcl@{}} {dY}_{t} & =&-\lambda. Y_{t}dt+{dZ}_{\lambda t}, \end{array} $$

where. denotes elementwise multiplication. Here, the coefficients of *S*_*t*_ are given by

$$\begin{array}{@{}rcl@{}} \mu_{t} & =&\mu_{0}+diag\left(\sigma_{t}\sigma_{t}^{\mathcal{T}}\right) \mu_{1}\\ \sigma_{t} & =&\Lambda\,\sqrt{Y_{t}}, \end{array} $$

where $\mu _{0},\mu _{1}\in \mathbb {R}^{n}$, *σ*_*t*_, is a *n*×*m* volatility matrix and *Λ* is a *n*×*m*×*k* tensor. The tensor product, $\Lambda \sqrt {Y_{t}}$, contracts the resulting tensor so that the last dimension is dropped.

For the tensor *Λ* three constraints are imposed; all elements are non-negative, the sum over the last index equals to one, and *Λy* is non-singular for all $y\in \mathbb {R}_{+}^{k}$.

By construction *Y*_*t*_ is a multidimensional non-Gaussian Ornstein Uhlenbeck process

(3) $$ Y_{t}=e^{\lambda t}.\left(Y_{0}+{\int_{0}^{t}}e^{-\lambda u}{dZ}_{\lambda u}\right),  $$

where. denotes elementwise multiplication and the jumps of the subordinator are suppressed by the rate of decay parameter *λ*=(*λ*_1_,...,*λ*_*k*_). Note also that the time of the subordinator, *Z*, is dilated by *λ* which makes the marginal distributions of *Y*_*t*_ independent of *λ*. This makes the statistical estimation of the model easier see e.g. (Barndorff-Nielsen and Shephard 2001) and (Lindberg 2007).

### 2.2 The utility function and the relative portfolio

The utility function reflects the investor’s attitude towards risk. Here we will consider exponential utility, *U*(*x*)=− exp(−*γx*), for the intensity parameter *γ*>0. Positive intensity entails that the investor is risk-averse. This is a fairly reasonable property for most investors, as one token gives more virtue to an investor with small funds than for a rich dito.

The (externally given) non-scaled benchmark portfolio, $\pi ^{b^{\prime }}$, is a self financing *n* dimensional adapted process of the portfolio weightings, in capital, for the benchmark index of interest. Analogously, the actual holdings in capital for the investor is denoted *π*^*p*^, which also is a a self financing *n* dimensional adapted process. The wealth of a portfolio is denoted $V^{\cdot }=(\cdot)^{\mathcal {T}} \mathbf 1$.

We now introduce the benchmark portfolio,

$$\pi^{b}= \frac{V^{\pi^{p}}}{V^{\pi^{b'}}}\pi^{b'}. $$

Note here that *π*^*b*^ is not a self-financing portfolio but is continuously set to have the same wealth as the investor’s portfolio, see (Korn and Lindberg 2013) for a rigorous argument.

Furthermore, the progressively measurable strategy *π*=*π*^*p*^−*π*^*b*^ is the relative holdings of the investor compared to the benchmark. We let *X*_*t*_ be the excess wealth of the portfolio above the benchmark at time *t*, subsequently denoted relative wealth. Then, by the stock price dynamics defined in equation (1), *X*_*t*_ is governed by

$${dX}_{t}=\pi_{t}^{\mathcal{T}}\left(\mu_{t}dt+\sigma_{t}{dW}_{t}\right), $$

where $\pi _{t}=\left (\pi _{t}^{(j)}\right)_{j=1,...,n}$ is such that $\pi _{t}^{(j)}$ denotes the relative amount of money invested in stock *j* at time *t*∈[ 0,*T*].

The set-up of relative portfolios is natural from a practicioner’s view point. In the financial industry, active portfolio managers are evaluated against a benchmark and strive to generate positive wealth in excess of that specific benchmark, so-called alpha. Hence, the relative portfolio is simply an industry chosen approach to evaluate performance. Here we give a brief justification of the set-up taken in this paper.

A naive way to measure the skill of an active portfolio manager would be to monitor the wealth of the investor’s portfolio minus the the wealth of the non-scaled benchmark, $V^{\pi ^{p}}-V^{\pi ^{b'}}$. In order to show that this is an inappropriate measure of skill for an active portfolio manager we introduce some additional notation; a bar over the portfolio denote the number of stocks in the portfolio, e.g., $\bar {\pi ^{p}}= \pi ^{p}./S_{t}$ where./ denotes elementwise division is the number of the respective stock in the investors portfolio. By the self-financing properties of the non-scaled benchmark and the investor’s portfolios the differential of $V^{\pi ^{p}}-V^{\pi ^{b'}}$ can be reformulated by the benchmark to equal

(4) $$  \begin{aligned} dV^{\pi^{p}}-dV^{\pi^{b'}}&= \left(\bar{\pi}_{t}^{p}\right)^{\mathcal{T}}{dS}_{t} -\left(\bar{\pi}_{t}^{b'}\right)^{\mathcal{T}}{dS}_{t} \\ &= \left(\bar{\pi}_{t}^{p}-\bar{\pi}_{t}^{b}\right)^{\mathcal{T}}{dS}_{t} +\left(\bar{\pi}_{t}^{b}-\bar{\pi}_{t}^{b'}\right)^{\mathcal{T}}{dS}_{t}, \end{aligned}  $$

where the first term is the relative wealth and the second term constitutes the beta effects. From equation (4) it is evident that the difference in wealth between the portfolio and the non-scaled benchmark depends on the overall market fluctations. In the financial industry, it is common to measure performance with zero net exposure to the market and relative wealth is the industry standard measure of skill for active portfolio managers.

### 2.3 The optimal value function

For some deterministic evaluation time *T*∈(0,*∞*), the *value functionJ* is defined as

$$J(t,x,y,\pi)=\mathbb{E}\left[U(X_{T})|X_{t}=x,Y_{t}=y,\pi\right], $$

for the relative portfolio *π*, current wealth *x* and news state *y*. Furthermore, *Γ* denotes the set of all relative strategies *π*=*π*^*p*^−*π*^*b*^ such that *π*^*p*^ is self-financing and for vectors $\mathbf {c}_{+},\mathbf {c}_{-}\in \mathbb {R}_{+}^{k}$, the relative money invested in stock *j* satisfies $-\mathbf {c}_{-}^{\left (j\right) }\leq \pi _{t} ^{(j)}\leq \mathbf {c}_{+}^{\left (j\right) }$ for all *j*=1,...,*n* and *t*∈[ 0,*T*]. The *optimal value functionV* is given by

(5) $$ V(t,x,y)=\sup_{\pi\in\Gamma}J(t,x,y,\pi),  $$

which is the investor’s maximum expected utility of terminal relative wealth. Furthermore a relative portfolio, *π*^∗^, which replicates a value function equal to the optimal value function such that *V*(*t*,*x*,*y*)=*J*(*t*,*x*,*y*,*π*^∗^) for all (*t*,*x*,*y*) is called the optimal portfolio.

## The control problem

Now, when we have the market model set up, we will in detail study the optimization problem. Our solution approach is to solve the Hamilton-Jacobi-Bellman (HJB) equation associated with equation (5).

For brevity, let the infinitesimal operator of the function *v*(*t*,*x*,*y*) be

$$ \begin{aligned} \mathcal{V}v & =\frac{\partial v}{\partial t}+\sup_{\pi\in\Gamma} \left(\pi^{\mathcal{T}}\mu\frac{\partial v}{\partial x}\,+\,\frac{1}{2}\pi ^{\mathcal{T}}\sigma\sigma^{\mathcal{T}}\pi\frac{\partial^{2}v}{\partial x^{2}}\right)\,-\,(\lambda.y)^{\mathcal{T}}\frac{\partial v}{\partial y}\\ & \quad+\sum_{i=1}^{k}\int_{0}^{\infty}v\left(t,x,y+{ze}_{i}\right)-v(t,x,y)m_{i}(dz), \end{aligned} $$

where *μ* and *σ* are functions of *y*, the. denotes element wise multiplication, *e*_*i*_ is the unity vector in the *i*th dimension, and *m*_*i*_ are the Lévy measures associated with the subordinators *Z*_*i*_.

The original optimization problem of Section 2 is now reformulated by Itô’s formula as finding a function, $v:[0,T]\times (0,\infty)^{k+1}\rightarrow \mathbb {R}$, such that

(6) $$\begin{array}{@{}rcl@{}} \mathcal{V}v&=&0\\ v(T,x,y)&=&U(x). \end{array} $$

Equation (6) is hereafter referred to as the HJB equation. We remark that, once the solution to the HJB equation is found a verification result is needed in order to guarantee that the solution coincides with the optimal value function, see e.g. (Korn and Kraft 2004).

## Well definedness of the optimal value function

In this section we will show that the optimal value function is well defined. This will be done by invoking two basic constraints of the model. Recall **c**_+_ and **c**_−_ are the upper and lower limits of *π*, see Section 2.3. First constraint; the constant *c*_*π*_= max(∥**c**_+_∥_*∞*_,∥**c**_−_∥_*∞*_) is bounded, recall this implies ∥*π*∥_*∞*_≤*c*_*π*_, i.e. there is a bound on the amount of relative wealth invested short and long in each stock. Second constraint; there exist a constant, $c_{L}\in \mathbb {R}$, such that for all *c*≤*c*_*L*_ then

(7) $$ \int_{0+}^{\infty}\left(e^{cz}-1\right){dm}_{i}(z)<\infty\quad\forall i=1,...,k  $$

which is equivalent to $\sum _{i=1}^{k}|\int _{0+}^{\infty }(e^{c_{L}z}-1){dm}_{i}(z)|<\infty $. The financial interpretation of the second condition is more complex. But equation (7) essentially gives a bound of the impact of news process jumps for the volatilities.

In order to derive the well definedness of the optimal value function two lemmas will be utilized.

### Lemma 1.

If equation (7) holds for every *c*≤*c*_*L*_ then for some $c_{1}\in \mathbb {R}$ we have $\sum _{i=1}^{k}\mathbb {E} \left [\exp \left (c_{1}{\int _{0}^{T}}{Y_{u}^{i}}du\right)\right ]<\infty $.

The proof of Lemma 1 rests on the coarse bound *Z*_*λt*_≤*Z*_*λT*_, where the inequality applies element-wise, for every *t*≤*T* and is due to that the subordinators are non-decreasing.

### **Proof of Lemma1**.

By equation (3) and the mentioned bound,

$$\begin{aligned} & \mathbb{E}\left[\exp\left(c_{1}{\int_{0}^{T}}{Y_{u}^{i}}du\right)|Y_{0}=y\right]\\ &\leq\mathbb{E}\left[\exp\left(c_{1}{\int_{0}^{T}}e^{\lambda_{i}u}\left(y_{i}+Z_{\lambda_{i}u}^{i}\right)du\right)\right]\\ & =e^{c_{1}\frac{e^{\lambda_{i}T}}{\lambda_{i}}y_{i}}\mathbb{E}\left[\exp\left(c_{1}{\int_{0}^{T}} e^{\lambda_{i}u}Z_{\lambda_{i}u}^{i}du\right)\right], \end{aligned} $$

where the term outside the expected value is finite. Furthermore, by another application of the same bound then

$$\begin{aligned} &\mathbb{E}\left[\exp(c_{1}{\int_{0}^{T}}e^{\lambda_{i}u}Z_{\lambda_{i}u}^{i}du\right] \\ &\leq\mathbb{E}\left[\exp\left(c_{1}Te^{\lambda_{i}T}Z_{\lambda_{i}T}^{i}\right)\right]\\ & =\exp\left(\lambda_{i}T\int_{0+}^{\infty}\left(e^{c_{1}Te^{\lambda_{i}T}z} -1\right){dm}_{i}(z)\right), \end{aligned} $$

where the equality is due to the infinite divisibility of Lévy processes, and thus $c_{1}Te^{||\lambda ||_{\infty }T}\leq c_{L}$ is a sufficient condition for

$$\sum_{i=1}^{k}\mathbb{E}\left[\exp\left(c_{1}{\int_{0}^{T}}{Y_{u}^{i}}du\right)|Y_{0}=y\right]<\infty. $$

The second lemma deals with the well definedness of $\mathbb {E}\left [\exp \left (c{\int _{0}^{T}}\left (\mathbf {1}^{\mathcal {T}}\sigma _{u}\sigma _{u}^{\mathcal {T}}\mathbf {1}\right)du\right)\right ]$ for some $c\in \mathbb {R}$. Recall that $\sigma _{u}=\Lambda \sqrt {Y_{u}}$ and let *Λ*=(*α*_*ijl*_) and

$$\alpha_{l,l^{\prime}}=\sum\limits_{i=1}^{n}\sum\limits_{i^{\prime}= 1}^{n} \sum\limits_{j=1}^{m} \alpha_{ijl} \alpha_{i^{\prime}jl^{\prime}} $$

so that

$$\mathbf{1}^{T}\sigma_{u}{\sigma_{u}^{T}}\mathbf{1}=\sum\limits_{l=1}^{k}{Y_{u}^{l}} \alpha_{l,l}+\sum\limits_{l=1}^{k}\sum\limits_{l^{\prime}\not =l}\sqrt{{Y_{u}^{l}} Y_{u}^{l^{\prime}}}\alpha_{l,l^{\prime}}. $$

### Lemma 2.

If

$$c_{2}\leq\frac{c_{L}}{Te^{||\lambda||_{\infty}T}\left(\max_{1\leq l\leq k} \sum_{l\not =l^{\prime}}\alpha_{l,l^{\prime}}+\max_{1\leq l\leq k}\alpha _{l,l}\right)}, $$

then we have

$$\mathbb{E}\left[\exp\left(c_{2}{\int_{0}^{T}}\left(\mathbf{1}^{\mathcal{T}}\sigma_{u}\sigma _{u}^{\mathcal{T}}\mathbf{1}\right)du\right)\right]<\infty. $$

### **Proof of Lemma2**.

By the definition of *σ*_*u*_,

(8) $$  \begin{aligned} &\mathbb{E}\left[e^{c_{2}{\int_{0}^{T}}\left(\mathbf{1}^{T}\sigma_{u}\sigma_{u}^{T}\mathbf{1}\right)du}\right]\\ &=\mathbb{E}\left[\exp\left(c_{2}\sum_{l=1}^{k}\alpha_{l,l}{\int_{0}^{T}}{Y_{u}^{l}}du\,+\,\sum_{l=1}^{k}\sum_{l^{\prime}\not =l}\alpha_{l,l^{\prime}}{\int_{0}^{T}}\sqrt{{Y_{u}^{l}}Y_{u}^{l^{\prime}}}du\right)\right]\\ &\leq\mathbb{E}\left[e^{{pc}_{2}\sum_{l=1}^{k}\alpha_{l,l}{\int_{0}^{T}}{Y_{u}^{l}} du}\right]^{\frac{1}{p}}\mathbb{E}\left[e^{{qc}_{2}\sum_{l=1}^{k}\sum_{l^{\prime}\not = l}\alpha_{l,l^{\prime}}{\int_{0}^{T}}\sqrt{{Y_{u}^{l}}Y_{u}^{l^{\prime}}} du}\right]^{\frac{1}{q}}, \end{aligned}  $$

where we have used Hölder’s inequality with conjugate exponents *p*,*q*. We begin by addressing the foremost term. By independence of the news processes,

(9) $$ \mathbb{E}\left[e^{{pc}_{2}\sum_{l=1}^{k}\alpha_{l,l}{\int_{0}^{T}}{Y_{u}^{l}}du} \right]=\Pi_{l=1}^{k}\mathbb{E}\left[e^{{pc}_{2}\alpha_{l,l}{\int_{0}^{T}}{Y_{u}^{l}}du}\right]  $$

and in addition by choosing

$$p=\frac{\max_{1\leq l\leq k}\sum_{l\not =l^{\prime}}\alpha_{l,l^{\prime}} +\max_{1\leq l\leq k}\alpha_{l,l}}{\max_{1\leq l\leq k}\alpha_{l,l}} $$

then *pc*_2_ max1≤*l*≤*kα*_*l*,*l*_≤*c*_1_ so that equation (9) is finite by a direct application of Lemma 1. For the latter term in equation (8), observe that $\sqrt {Y^{l}Y^{l^{\prime }}}\leq (Y^{l}+Y^{l^{\prime }})/2$ and by a double application of independence of different news processes then we have

$$\begin{aligned} &\mathbb{E}\left[e^{{qc}_{2}\sum_{l=1}^{k}\sum_{l^{\prime}\not =l}\alpha_{l^{\prime },l}{\int_{0}^{T}}\sqrt{{Y_{u}^{l}}Y_{u}^{l^{\prime}}}du}\right]\\ &\leq\mathbb{E}\left[e^{\frac{{qc}_{2}}{2}\sum_{l=1}^{k}\sum_{l^{\prime}\not =l}\alpha_{l,l^{\prime }}{\int_{0}^{T}}{Y_{u}^{l}}+Y_{u}^{l^{\prime}}du}\right]\\ &=\mathbb{E}\left[e^{\frac{{qc}_{2}}{2}\sum_{l=1}^{k}\sum_{l^{\prime}\not = l}2\alpha_{l,l^{\prime}}{\int_{0}^{T}}{Y_{u}^{l}}du}\right]\\ &=\mathbb{E}\left[e^{{qc}_{2}\sum_{l=1}^{k}\sum_{l^{\prime}\not =l}\alpha_{l,l^{\prime}}{\int_{0}^{T}}{Y_{u}^{l}}du}\right]\\ &=\Pi_{l=1}^{k}\mathbb{E}\left[e^{{qc}_{2}\sum_{l^{\prime}\not =l}\alpha_{l,l^{\prime}}{\int_{0}^{T}}{Y_{u}^{l}}du}\right]. \end{aligned} $$

This is bounded by Lemma 1 since the conjugate exponent

$$q=\frac{\max_{1\leq l\leq k}\sum_{l\not =l^{\prime}}\alpha_{l,l^{\prime}} +\max_{1\leq l\leq k}\alpha_{l,l}}{\max_{1\leq l\leq k}\sum_{l\not =l^{\prime }}\alpha_{l,l^{\prime}}}, $$

so that ${qc}_{2}\max _{1\leq l\leq k}\sum _{l^{\prime }\not =l}\alpha _{l,l^{\prime }}\leq c_{1}$.

Now are we ready to state and prove the well definedness of the optimal value function.

### Proposition 1.

If there exist a big enough constants $c_{\pi },c_{L} \in \mathbb {R}$ such that ||*π*||_*∞*_≤*c*_*π*_ and equation (7) holds for every *c*≤*c*_*L*_ then $\mathbb {E}\left [|U\left (X_{T}\right)|\mid X_{t}=x,Y_{t}=y,\pi \right ]$ is finite.

Before we start, let $\xi =-\gamma \left (X_{t}+{\int _{t}^{T}}\pi ^{\mathcal {T}}\mu _{u}du+c_{\pi }\right.{\int _{t}^{T}}\mathbf {1}^{\mathcal {T}}\left.\vphantom {{\int _{t}^{T}}}\sigma _{u}{dW}_{u}\right)$. We will see that

(10) $$ \mathbb{E}\left[\exp(\xi)|X_{t}=x,Y_{t}=y,\pi\right]<\infty  $$

is a sufficient condition for the optimal value function to be well defined. Assume that equation (10) holds. Then we have by Jensen’s inequality that $\mathbb {E}[\xi ]$ exists. Note that *ξ* and −*γX*_*T*_ share first moment while *P*(*ξ*>*x*)≥*P*(−*γX*_*T*_>*x*) for all $x\geq \mathbb {E}[\xi ]$, where probabilities are conditional *X*_*t*_=*x*,*Y*_*t*_=*y* and *π*. Now, with *P*_*ξ*_ denoting the distribution function of *ξ*,

$$ \begin{aligned} &\mathbb{E}\left[e^{\max(\xi-\mathbb{E}[\xi],0)}\right]\\ &=\sum_{k=0}^{\infty}{\frac{\mathbb{E}\left[\max(\xi-\mathbb{E}[\xi],0)^{k}\right]}{k!}}\\ &=\sum_{k=0}^{\infty}{\frac{\int_{\mathbb{E}[\xi]}^{\infty}\left(x-\mathbb{E} [\xi]\right)^{k}{dP}_{\xi}(x)}{k!}}\\ &=P(\xi\geq\mathbb{E}[\xi])+\sum_{k=1}^{\infty}{\frac{\int_{\mathbb{E}[\xi ]}^{\infty}(x-\mathbb{E}[\xi])^{k-1}P(\xi>x)dx}{k!}}\\ &\geq P\left(-\gamma X_{T}\geq\mathbb{E}[\xi]\right)\,+\,\sum_{k=1}^{\infty}{\frac {\int_{\mathbb{E}[\xi]}^{\infty}(x\,-\,\mathbb{E}[\xi])^{k-1}P(-\gamma X_{T} >x)dx}{k!}}\\ &=\mathbb{E}\left[e^{\max\left(-\gamma X_{T}-\mathbb{E}[\xi],0\right)}\right], \end{aligned} $$

where we have used Tonelli-Fubini twice, see e.g. (Durrett 2010). Further,

$$ \begin{aligned} \mathbb{E}\left[e^{\xi}\right]+e^{\mathbb{E}[\xi]}&\geq e^{\mathbb{E}[\xi]}\mathbb{E} \left[e^{\max(\xi-\mathbb{E}[\xi],0)}\right]\\ &\geq e^{\mathbb{E}[\xi]}\mathbb{E} \left[e^{\max(-\gamma X_{T}-\mathbb{E}[\xi],0)}\right]\geq\mathbb{E}\left[e^{-\gamma X_{T}}\right] \end{aligned} $$

and the desired result follows.

We will need an auxiliary function in the proof, to bound the integrals involving the Brownian motions for the optimal value function. For *s*≥*t*, let

$$\Psi_{s}=2\gamma c_{\pi}{\int_{t}^{s}}\mathbf{1}^{\mathcal{T}}\sigma_{u}{dW}_{u}. $$

Further, by an application of Lemma 2 then if *c*_2_≥2(*γc*_*π*_)^2^, we have that

$$\mathbb{E}\left[\exp\left(2\left(\gamma c_{\pi}\right)^{2}{\int_{t}^{T}} \mathbf{1}^{\mathcal{T}}\sigma_{u}\sigma_{u}^{\mathcal{T}}\mathbf{1} du\right)\right]<\infty. $$

In addition, Novikov’s condition, see e.g. (Protter 2010)[theorem 45], gives that $\mathcal {E}(\Psi _{s})$ is a martingale and thus has constant expected value equal to one, since *Ψ*_0_=0.

### **Proof of Lemma3**.

By definition of *ξ* and the martingale $\mathcal {E}(\Psi _{s})$ then,

(11) $$  {\small{\begin{aligned} &\mathbb{E}[e^{\xi}]\\ & =\mathbb{E}\left[\exp\left(-\gamma\left(X_{t} +{\int_{t}^{T}}\pi^{\mathcal{T}}\mu_{u}du+c_{\pi}{\int_{t}^{T}}\mathbf{1} ^{\mathcal{T}}\sigma_{u}{dW}_{u}\right) \right) \right]\\ &=e^{-\gamma x}\mathbb{E}\left[\exp\left(-{\gamma\int_{t}^{T}}\pi^{\mathcal{T} }\mu_{u}du-c_{\pi}{\gamma\int_{t}^{T}}\mathbf{1}^{\mathcal{T}}\sigma_{u} {dW}_{u}\right) \right]\\ &=e^{-\gamma x}\mathbb{E}\left[\exp\left(-{\gamma\int_{t}^{T}}\pi^{\mathcal{T} }\mu_{u}du+\frac{\left(\gamma c_{\pi}\right)^{2}}{2}{\int_{t}^{T}} \mathbf{1}^{\mathcal{T}}\sigma_{u}\sigma_{u}^{\mathcal{T}}\mathbf{1}du\right)\right.\\ &\quad\times\left. \vphantom{\int_{t}^{T}}\mathcal{E}(\Psi_{T})^{\frac{1}{2}}\right]\\ &\leq e^{-\gamma x}\mathbb{E}\left[\exp\left(2\left\vert {\int_{t}^{T}}-\gamma\pi^{T} \mu_{u}+\frac{(c_{\pi}\gamma)^{2}}{2}\mathbf{1}^{T}\sigma_{u}\sigma_{u}^{T}\mathbf{1}du\right\vert \right)\right]^{\frac{1}{2}}\\ &\quad\times\mathbb{E}[\mathcal{E}(\Psi_{T})]^{\frac{1}{2}}\\ &\leq e^{-\gamma x}\mathbb{E}\left[\exp\left(2{\int_{t}^{T}}\gamma c_{\pi}\mathbf{1}^{T}(||\mu^{0}||_{\infty}+\sigma_{u}{\sigma_{u}^{T}}\mathbf{1}||\mu^{1}||_{\infty})\right.\right.\\ &\quad+\left.\left.\vphantom{\int_{t}^{T}}\frac{(c_{\pi}\gamma)^{2}}{2}\mathbf{1}^{T}\sigma_{u}{\sigma_{u}^{T}}\mathbf{1}du\right)\right]^{\frac{1}{2}}\\ & \leq e^{-\gamma x+2c_{\pi}\gamma||\mu_{0}||_{\infty}T}\mathbb{E} \left[e^{(c_{\pi}\gamma\vee1)^{2}(2||\mu^{1}||_{\infty}+1){\int_{0}^{T}} \left(\mathbf{1}^{T}\sigma_{u}{\sigma_{u}^{T}}\mathbf{1}\right)du}\right]^{\frac{1}{2}}, \end{aligned}}}  $$

where first we have used an application of the Cauchy-Schwarz inequality and secondly the definition of the appreciation rate *μ*. Furthermore, if

$$c_{2}\geq\left(c_{\pi}\gamma\vee1\right)^{2}\left(2||\mu^{1}||_{\infty}+1\right), $$

then by an application of Lemma 2, the right-hand side of equation (11) is finite which yields the desired result.

## Verification theorem

Here, we will prove that the well defined solution to the HJB equation is a maximizer of the value function and thus a solution to the optimization problem. This section follows the deduction in (Korn and Lindberg 2013).

### Theorem 1 (Verification Theorem).

Suppose $v(t,x,y)\in C^{1,2,\mathbf {1}^{\mathcal {T}}}\left ([0,T]\times (0,\infty)^{k+1}\right)$, where $\mathbf {1}^{\mathcal {T}}$ is of length *k*, is a solution to the HJB equation given in equation (6). Also let,

(12) $$  \sup_{\pi\in\Gamma}{\int_{0}^{T}}\mathbb{E}\left[v_{x}(t,X_{t},Y_{t-})^{2} \pi^{\mathcal{T}}\sigma_{t}\sigma_{t}^{\mathcal{T}}\pi\mid X_{0} \,=\,x,Y_{0}\,=\,y\right]dt<\infty,  $$

where

$$ \begin{aligned} \Gamma&=\left\{\pi=\pi^{p}-\pi^{b}:\pi_{t}^{\mathcal{T}}\mathbf{1} =0\ \text{and}\ -\mathbf c_{-}^{\left(j\right) }\leq\pi_{t}^{(j)}\leq \mathbf{c}_{+}^{(j)},\right.\\ &\,\,\quad\forall\left.\vphantom{\mathbf{c}_{+}^{(j)}} j=1,...,n\ \text{and}\ t\in[0,T]\right\} \end{aligned} $$

and *π*^*p*^ is a self financing portfolio and *π*^*b*^ the corresponding benchmark portfolio, and

(13) $$  \begin{aligned} &\sum_{i=1}^{k}{\int_{0}^{T}}\int_{0+}^{\infty}\mathbb{E}\left[|v(t,X_{t},Y_{t-}+{ze}_{i})-v(t,X_{t},Y_{t-})|\mid X_{0}\,=\,x,\right.\\ &\qquad\qquad\qquad\left.Y_{0}=y\right]{dm}_{i}(z)dt<\infty \end{aligned}.  $$

Then,

(14) $$ v(t,x,y)\geq V(t,x,y),\quad\forall(t,x,y)\in[0,T]\times\mathbb{R} _{+}^{k+1}.  $$

Further, if there exist a measurable admissible trading strategy, *π*^∗^, such that *J*(*t*,*x*,*y*,*π*^∗^)=*v*(*t*,*x*,*y*) then that trading strategy is the optimal trading strategy for the portfolio optimization problem of maximizing utility of relative wealth in the B-NS market.

For brevity, let the infinitesimal operator of *v*(*t*,*x*,*y*) given portfolio *π* be

$$\begin{array}{@{}rcl@{}} \mathcal{V}_{\pi}v & =&\frac{\partial v}{\partial t}+\pi^{\mathcal{T}} \mu\frac{\partial v}{\partial x}+\frac{1}{2}\pi^{\mathcal{T}}\sigma \sigma^{\mathcal{T}}\pi\frac{\partial^{2}v}{\partial x^{2}}-(\lambda.y)^{\mathcal{T}}\frac{\partial v}{\partial y}\\ && +\sum\limits_{i=1}^{k}\int_{0}^{\infty}v(t,x,y+z\cdot e_{i})-v(t,x,y)m_{i} (dz), \end{array} $$

where in accordance with previous notation. denotes element-wise multiplication, $\frac {\partial v}{\partial y}$ is of *k*-dimension and *e*_*i*_ denotes the unity vector of *i*th dimension.

### **Proof of Lemma4**.

By Itō’s formula, equation (12), and equation (13), then

$$ \begin{aligned} &J(t,x,y,\pi)\\ & =\mathbb{E}[U(X_{T})\mid X_{t}=x,Y_{t}=y,\pi]\\ &=v(t,x,y)\,+\,\mathbb{E}\left[{\int_{t}^{T}}\mathcal{V}_{\pi}v(u,X_{u},Y_{u})du\mid X_{t}\,=\,x,Y_{t}\,=\,y\right]\\ &\leq v(t,x,y)\,+\,\mathbb{E}\left[{\int_{t}^{T}}\sup_{\pi\in\Gamma}\mathcal{V}_{\pi }v(u,X_{u},Y_{u})du\mid X_{t}\,=\,x,Y_{t}\,=\,y\right]\\ &=v(t,x,y), \end{aligned} $$

which is the first part of the theorem. Further, with the optimal portfolio *π*^∗^ then

$$\sup_{\pi\in\Gamma}\mathcal{V}_{\pi}v=\mathcal{V}_{\pi^{\ast}}v $$

which yields *J*(*t*,*x*,*y*,*π*^∗^)=*v*(*t*,*x*,*y*) and thus is *v*(*t*,*x*,*y*) the solution to the optimization problem and *π*^∗^ is the optimal portfolio in the setting of the B-NS factor dynamics.

## Probabilistic representation of the solution

Here a probabilistic representation of the solution to the HJB equation will be given. By suitable conjectures, the original HJB equation is first reduced and subsequently solved. The solution is of Feynman-Kac form.

By (Korn and Lindberg 2013), we make the ansatz that the optimal value function *v*(*t*,*x*,*y*)=−*e*^−*γx*^*h*(*t*,*y*), for some function *h*. Again for brevity, let the infinitesimal operator of *h*(*t*,*y*) be

$$ \begin{aligned} \mathcal{H}h & =\frac{\partial h}{\partial t}+h(t,y)\gamma\sup_{\pi\in \Gamma}\left\{\pi^{\mathcal{T}}\mu-\frac{\gamma}{2}\pi^{\mathcal{T}}\sigma \sigma^{\mathcal{T}}\pi\right\}\\ &\quad-(\lambda.y)^{\mathcal{T}}\frac{\partial h}{\partial y}+\sum_{i=1}^{k}\int_{0}^{\infty}h(t,y+{ze}_{i})-h(t,y)m_{i}(dz), \end{aligned} $$

with previously introduced notation for. and *e*_*i*_. By the postulated function, the original HJB equation is reduced to finding a function $h:[0,T]\times (0,\infty)^{k}\rightarrow \mathbb {R}$, such that

(15) $$\begin{array}{@{}rcl@{}} \mathcal{H}h&=&0\\ h(T,\cdot)&=&1. \end{array} $$

We will start out by defining a function *h*(*t*,*y*) and by some effort it will become clear that the proposed function solves equation (15). In order to ease up the computations some further notation is introduced. Let $\Pi :\mathbb {R}_{+}^{k}\rightarrow \mathbb {R}_{+}$ and *ω*:*D*^*k*^→*C*^1^[0,*T*], where *D* denotes the Skorohod space and thus *D*^*k*^ consists of *k*-dimensional cádlág functions, be such that

$$\Pi(y)=\gamma\sup_{\pi\in\Gamma}\left\{\pi^{\mathcal{T}}\mu-\frac{\gamma}{2} \pi^{\mathcal{T}}\sigma\sigma^{\mathcal{T}}\pi\right\} $$

and

$$\omega_{\cdot}(Y)=\int_{0}^{\cdot}\Pi(Y_{u})du. $$

We state now a series of lemmas which characterizes the properties of *Π* and *ω*. These are proved in Section 6.1.

### Lemma 3.

*Π*(*y*) is non negative and well defined for all $y\in \mathbb {R}_{+}^{k}$.

For the following two lemmas the stochastic dynamics of *Y* is given by equation (2).

### Lemma 4.

For all *τ*∈[0,*T*] and $y\in \mathbb {R} _{+}^{k}$,

$$\mathbb{E}\left[e^{2\omega_{\tau}(Y)}|Y_{0}=y\right]<\infty. $$

### Lemma 5.

For all *τ*∈[ 0,*T*], $y\in \mathbb {R}_{+}^{k} $,

$$\sum_{i=1}^{k}\int_{0}^{\infty}\mathbb{E}\left[e^{\omega_{\tau}(Y)}|Y_{0} \,=\,y\,+\,{ze}_{i}\right]-\mathbb{E}\left[e^{\omega_{\tau}(Y)}|Y_{0}\,=\,y\right]{dm}_{i}(z)<\infty. $$

Note that by an application of Jensen’s inequality then Lemma 4 entails that $\mathbb {E}\left [e^{\omega _{\tau } (Y)}|Y_{0}=y\right ]$ is well defined. We assume now that

(16) $$ h(t,y)=\mathbb{E}\left[e^{\omega_{T}(Y)-\omega_{t}(Y)}|Y_{t}=y\right]  $$

is a solution to the reduced HJB equation, equation (15) and state an additional lemma:

### Lemma 6.

For all *τ*∈[0,*T*], $y\in \mathbb {R}_{+}^{k}$, ∇_*y*_*h* exists and is continuous.

Note that the stochastic process, $\phantom {\dot {i}}t\mapsto e^{\omega _{T}(Y)-\omega _{t}(Y)}$, features the Markov property and thus $h(t,y)=\mathbb {E}\left [e^{\omega _{T-t} (Y)}|Y_{0}=y\right ]$ i.e. the expected exponential of *ω*_*T*−*t*_(*Y*) conditional on the initial value of *Y* set at time *t*=0. Further, by Lemma 4 with *τ*=*T*−*t*, we have that *h*(*t*,*y*) is well defined, and since *h*(*T*,·)=1, the terminal condition of the associated HJB equation is satisfied by the proposed function.

By Lemma 4 and dominated convergence, see e.g. (Folland 1999)[theorem 2.27], we are allowed to arbitrary shift order of limits and expected values regarding the function $\phantom {\dot {i}}e^{\omega _{\cdot }(Y)}$. By this property then $h_{t}^{\prime }(t,y)$ is well defined since;

(17) $$\begin{array}{@{}rcl@{}} h_{t}^{\prime}(t,y) & =&\mathbb{E}\left[\frac{d}{dt}e^{\omega_{T-t}(Y)}\mid Y_{0}=y\right]\\ & =&\mathbb{E}\left[-\omega_{t}^{\prime}(Y)e^{{\int_{t}^{T}}\Pi(Y_{u})du}\mid Y_{t}=y\right]\\ & =&-\Pi(y)h(t,y), \end{array} $$

where *Π*(*y*) and *h*(*t*,*y*) are finite by Lemmas 3 and 4. Note, by equation (17), the interesting property that the ratio $h_{t}^{\prime }(t,y)/h(t,y)$ is independent of *t*, a property that will be utilized below.

Next, for a fixed *τ*∈[ 0,*T*] and by the aid of the auxiliary function $g_{\tau }:\mathbb {R}_{+}^{k}\rightarrow \mathbb {R}$ such that $g_{\tau }(y)=h(T-\tau,y)=\mathbb {E}\left [e^{\omega _{\tau }(Y)}|Y_{0}=y\right ]$, we will see that *h*(*t*,*y*) solves the reduced HJB equation. Furthermore, for *ε*>0, we have

(18) $$\begin{array}{@{}rcl@{}} \mathbb{E}[{g_{\tau}(Y_{\epsilon})}]\mid Y_{0}=y]&=&\mathbb{E} \left[\mathbb{E}\left[e^{\omega_{\tau}(Y)}\mid Y_{0}=Y_{\epsilon}\right]\mid Y_{0} =y\right]\\ &=&\mathbb{E}\left[\mathbb{E}\left[e^{\int_{0}^{\tau}\Pi(Y_{u+\epsilon})du} \mid\mathcal{F}_{\epsilon}\right]\mid\mathcal{F}_{0}\right]\\ & =&\mathbb{E}\left[\mathbb{E}\left[e^{\int_{\epsilon}^{\tau+\epsilon}\Pi(Y_{u})du} \mid\mathcal{F}_{\epsilon}\right]\mid\mathcal{F}_{0}\right]\\ & =&\mathbb{E}\left[e^{\int_{\epsilon}^{\tau+\epsilon}\Pi(Y_{u})du}\mid \mathcal{F}_{0}\right]\\ & =&\mathbb{E}\left[e^{\omega_{\tau+\epsilon}(Y)-\omega_{\epsilon}(Y)}\mid Y_{0}=y\right], \end{array} $$

where the first equality is due to the definition of *g* and the second equality is due to the Markov property. By equation (18) and the non-negativeness of *Π*

$$\mathbb{E}[{g_{\tau}(Y_{\epsilon})]}\mid Y_{0}=y]\leq\mathbb{E}\left[e^{\omega _{2T}(Y)}\mid Y_{0}=y\right], $$

sufficiently small *ε*. Further, by a variable substitution then $\omega _{2T}(Y)\stackrel {\mathcal {L}}{=}2\omega _{T}(Y)$, where $\stackrel {\mathcal {L}}{=}$ denotes equality in distribution and $\mathbb {E} [{g_{\tau }(Y_{\epsilon })]}\mid Y_{0}=y]$ is well defined by Lemma 4.

Now, by reversing the order of expectations and derivatives for *g*, we can show that the function *h* solves the reduced HJB equation (15). By an application of equation (18), for *ε*>0, we have that

$$\begin{aligned} & \mathbb{E}\left[{\frac{g(Y_{\epsilon})-g(y)}{\epsilon}}\mid Y_{0}=y\right]\\ &=\frac{1}{\epsilon}\mathbb{E}\left[e^{\omega_{\tau+\epsilon}(Y)-\omega_{\epsilon}(Y)}-e^{\omega_{\tau}(Y)}\mid Y_{0}=y\right]\\ &=\mathbb{E}\left[e^{\omega_{\tau+\epsilon}(Y)}\frac{1}{\epsilon}\left(e^{-\omega_{\epsilon}(Y)}-1\right)\mid Y_{0}=y\right]\\ &\quad+\frac{1}{\epsilon}\mathbb{E}\left[e^{\omega_{\tau+\epsilon}(Y)}-e^{\omega_{\tau}(Y)}\mid Y_{0}=y\right]\\ &=I_{1}+I_{2}, \end{aligned} $$

where

$$\begin{aligned} I_{1}&=\mathbb{E}\left[e^{\omega_{\tau+\epsilon}(Y)}\frac{1}{\epsilon }\left(e^{-\omega_{\epsilon}(Y)}-1\right)\mid Y_{0}=y\right]\\ & \rightarrow_{\epsilon}\mathbb{E}\left[e^{\omega_{\tau}(Y)}\left(-\omega_{\epsilon }^{\prime}(Y)\mid_{\epsilon=0}\right)\mid Y_{0}=y\right]\\ & =h(T-\tau,y)(-\Pi(y)) \end{aligned} $$

and

$$\begin{aligned} I_{2} & =\frac{1}{\epsilon}\mathbb{E}\left[e^{\omega_{\tau+\epsilon}(Y)} -e^{\omega_{\tau}(Y)}\mid Y_{0}=y\right]\\ & ={\frac{h(T-\tau+\epsilon,y)-h(T-\tau,y)}{\epsilon}}\\ & \rightarrow_{\epsilon}-h_{t}^{\prime}(T-\tau,y). \end{aligned} $$

Putting things together, by equation (17), *I*_1_+*I*_2_→_*ε*_0 and thus$\mathbb {E}[\frac {d}{dt}g_{\tau }(Y_{t})\mid Y_{0}=y]=0$. Furthermore, by an application of Itō’s formula for *g*_*τ*_(*Y*_*ε*_) with *ε*>0, then

(19) $$  \begin{aligned} 0 & \leftarrow_{\epsilon}\mathbb{E}\left[{\frac{g_{\tau}(Y_{\epsilon})-g_{\tau}(y)}{\epsilon}}\mid Y_{0}=y\right]\\ & =\mathbb{E}\left[-\frac{1}{\epsilon}\left({\vphantom{\sum_{i=1}^{k}}} \int_{0}^{\epsilon}(\lambda.Y_{u})^{\mathcal{T}}\frac{\partial g_{\tau}}{\partial y}(Y_{u})du-\sum _{i=1}^{k}\int_{0}^{\epsilon}\int_{0+}^{\infty}\right.\right. \\ &\quad\times\left.\left.{\vphantom{\sum_{i=1}^{k}}} g_{\tau}(Y_{u}+{ze}_{i})-g_{\tau }(Y_{u}){dm}_{i}(z)ds \right) \mid Y_{0}=y\right]\\ & \rightarrow_{\epsilon}-(\lambda.y)^{\mathcal{T}}\frac{\partial g_{\tau} }{\partial y}(y)+\sum_{i=1}^{k}\int_{0+}^{\infty}g_{\tau}(y+{ze}_{i})-g_{\tau }(y){dm}_{i}(z)\\ & =-(\lambda.y)^{\mathcal{T}}\frac{\partial h}{\partial y}(T-\tau,y)+\sum_{i=1}^{k}\int_{0+}^{\infty}h(T-\tau,y+{ze}_{i})\\ &\quad-h(T-\tau,y){dm}_{i} (z),\\ \end{aligned}  $$

where the last line is familiar from the reduced HJB equation. Since $\lambda,y\in \mathbb {R}_{+}^{k}$, by an application of Lemma 5 we have that $\frac {\partial h}{\partial y}(t,y)$ is well defined. To sum up, by applying equations (17) and (19) with *τ*=*T*−*t*, we have that $h(t,y)\in C^{1,\mathbf {1}^{\mathcal {T}} }\left ([0,T]\times \mathbb {R}_{+}^{k}\right)$ is a solution to the reduced HJB equation (15). Furthermore,

(20) $$ v(t,x,\sigma)=-\mathbb{E}\left[{e^{\omega_{T-t}(Y)-\gamma x}\mid Y_{0} =y}\right],  $$

is a well defined solution to the original HJB equation if Lemmas 3, 4, 5, and 6 holds.

### 6.1 Exposition of *Π*(*y*), *ω*_·_ and conjectured lemmas

In this section, the Lemmas 3, 4, 5, and 6 are proved, which are the cornerstones in showing the well definedness of the probabilistic representation of the solution to the HJB equation. We start out with a short detour to the function *Π*(*y*) where the exposition follows the deduction done in (Korn and Lindberg 2013). Next, Lemma 4 is proved with the aid of a linear growth condition implicit in the definition of *Π*. Lemma 5 follows by pursuing the growth condition combined with some properties of *Π*.

We will now analyze *Π*(*y*). Let $f\left (\pi \right) :=\pi ^{\mathcal {T}} \mu -\frac {\gamma }{2}\pi ^{\mathcal {T}}\sigma \sigma ^{\mathcal {T}}\pi $, and recall that

$$\Pi(y)=\gamma\sup_{\pi\in\Gamma}f\left(\pi\right), $$

where both *μ* and *σ* are functions of *y* and

$$ \begin{aligned} &\Gamma=\left\{\pi=\pi^{p}-\pi^{b}:\pi_{t}^{\mathcal{T}}\mathbf{1} =0\text{ and }-\mathbf c_{-}^{\left(j\right) }\leq\pi_{t}^{(j)}\leq \mathbf c_{+}^{\left(j\right) },\right.\\ &\qquad\left.\vphantom{c_{+}^{\left(j\right) }}\forall j=1,...,n\ \text{and}\ t\in[0,T]\right\} \end{aligned} $$

where *π*^*p*^ is a self financing portfolio and *π*^*b*^ the corresponding benchmark portfolio.

We solve this optimization problem by introducing the Karuch-Kuhn-Tucker multipliers, see e.g. (Nash and Sofer 1996), $\delta \in \mathbb {R}$ for the equality constraint, and $\nu _{+},\nu _{-}\in (\mathbb {R}_{+}\cup \{0\})^{n}$ for the upper and lower constraints of *π*, respectively. The optimal portfolio then satisfies

(21) $$\begin{array}{@{}rcl@{}} \mu-\gamma\sigma\sigma^{\mathcal{T}}\pi-\delta-\nu_{+}-\nu_{-} & =&\mathbf{0}\\ \pi^{\mathcal{T}}\mathbf{1} & =&0\\ \nu_{+}.(\pi-\mathbf{c}_{+}) & =&\mathbf{0} \end{array} $$

(22) $$\begin{array}{@{}rcl@{}} \nu_{-}.(\pi+\mathbf{c}_{-}) & =&\mathbf{0} \end{array} $$

where. denotes elementwise multiplication. These equations have the solution

(23) $$\begin{array}{@{}rcl@{}} \pi^{\ast} & =&\frac{1}{\gamma}\left(\sigma\sigma^{\mathcal{T}}\right)^{-1}\left( \mu\,-\,\nu_{+}^{\ast}\,-\,\nu_{-}^{\ast}\,+\,{\frac{\left(\mu\,-\,\nu_{+}^{\ast}\,-\,\nu_{-}^{\ast }\right)^{\mathcal{T}}\left(\sigma\sigma^{\mathcal{T}}\right)^{-1}\mathbf{1}}{\mathbf{1} ^{\mathcal{T}}\left(\sigma\sigma^{\mathcal{T}}\right)^{-1}\mathbf{1}}}\right)  \end{array} $$

(24) $$\begin{array}{@{}rcl@{}} \delta^{\ast} & =&{\frac{\left(\mu-\nu_{+}^{\ast}-\nu_{-}^{\ast}\right)^{\mathcal{T} }\left(\sigma\sigma^{\mathcal{T}}\right)^{-1}\mathbf{1}}{\mathbf{1}^{\mathcal{T}} \left(\sigma\sigma^{\mathcal{T}}\right)^{-1}\mathbf{1}}} \end{array} $$

where $\nu _{+}^{\ast },\nu _{-}^{\ast }$ satisfies equations (21) and (22). If none of the inequality constraints are sharp then we have that *ν*_+_=*ν*_−_=**0** and plugging *π*^∗^ in *f*(·), and noting that

$$\begin{aligned} & \left(\mu-{\frac{\mu^{\mathcal{T}}\left(\sigma\sigma^{\mathcal{T}}\right)^{-1}\mathbf{1} }{\mathbf{1}^{\mathcal{T}}\left(\sigma\sigma^{\mathcal{T}}\right)^{-1}\mathbf{1}} }\right)^{\mathcal{T}}\left(\sigma\sigma^{\mathcal{T}}\right)^{-1}{\frac{\mu^{\mathcal{T} }\left(\sigma\sigma^{\mathcal{T}}\right)^{-1}\mathbf{1}}{\mathbf{1}^{\mathcal{T}} \left(\sigma\sigma^{\mathcal{T}}\right)^{-1}\mathbf{1}}}\mathbf{1}\\ &=\gamma{\frac {\mu^{\mathcal{T}}\left(\sigma\sigma^{\mathcal{T}}\right)^{-1}\mathbf{1}}{\mathbf{1} ^{\mathcal{T}}\left(\sigma\sigma^{\mathcal{T}}\right)^{-1}\mathbf{1}}}\pi^{\ast \mathcal{T}}\mathbf{1}=0, \end{aligned} $$

we find that

(25) $$ \Pi(y)=\frac{1}{2}\left(\mu-{\frac{\mu^{\mathcal{T}}\left(\sigma\sigma^{\mathcal{T} }\right)^{-1}\mathbf{1}}{\mathbf{1}^{\mathcal{T}}\left(\sigma\sigma^{\mathcal{T}} \right)^{-1}\mathbf{1}}}\right)^{\mathcal{T}}\left(\sigma\sigma^{\mathcal{T}}\right)^{-1} \mu.  $$

Note that *Π*(*y*) is independent of the risk aversion parameter *γ* in this particular case. This does not hold in general.

In order to progress, we need bounds on *Π*(*y*). By definition of *Λ*, $\sigma \sigma ^{\mathcal {T}}$ is positive definite. Now we are ready to prove Lemma 3.

#### **Proof of Lemma5**.

The foremost part, *Π*(*y*)≥0, follows direct since the relative portfolio *π*=**0** is in *Γ*. The latter part follows by the definition of *f* combined with ∥*π*∥_*∞*_≤*c*_*π*_ and the positive definiteness of $\sigma \sigma ^{\mathcal {T}}$ so we have that *Π*(*y*)≤*c*_*π*_||*μ*||. Thus,

(26) $$ 0\leq\Pi(y)\leq c_{\pi}\left\Vert \mu_{0}\right\Vert_{\infty}+c_{\pi }\left\Vert \mu_{1}\right\Vert_{\infty}\mathbf{1}^{\mathcal{T}} y.  $$

Further, recall that $\omega _{\cdot }(Y)=\int _{0}^{\cdot }\Pi (Y_{u})du$. We are now equipped to prove Lemma 4.

#### **Proof of Lemma6**.

Since *Π* is non-negative, *ω*_·_ is non-negative and for *τ*∈[0,*T*]

$$ \omega_{\tau}(Y){\leq\int_{0}^{T}}\Pi(Y_{u})du\leq {Tc}_{\pi}\left\Vert \mu _{0}\right\Vert_{\infty}+c_{\pi}\left\Vert \mu_{1}\right\Vert_{\infty} {\int_{0}^{T}}\mathbf{1}^{\mathcal{T}}Y_{u}du, $$

and hence also *ω*_·_(*Y*) follows a linear growth condition. Further by applying these inequalities,

$$\begin{aligned} & \mathbb{E}\left[e^{2\omega_{\tau}(Y)}|Y_{0}=y\right]\\ & \leq e^{2{Tc}_{\pi}\left\Vert \mu_{0}\right\Vert_{\infty}}\mathbb{E}\left[e^{2c_{\pi}\left\Vert \mu _{1}\right\Vert_{\infty}\sum_{i=1}^{k}{\int_{0}^{T}}{Y_{u}^{i}}du}|Y_{0}=y\right]\\ & =e^{2{Tc}_{\pi}\left\Vert \mu_{0}\right\Vert_{\infty}}\Pi_{i=1} ^{k}\mathbb{E}\left[e^{2c_{\pi}\left\Vert \mu_{1}\right\Vert_{\infty}\int_{0} ^{T}{Y_{u}^{i}}du}|Y_{0}=y\right] \end{aligned} $$

where, by an application of Lemma 1, a sufficient condition for $\mathbb {E}\left [e^{2\omega _{\tau }(Y)}|Y_{0}=y\right ]$ to be finite is 2*c*_*π*_∥*μ*_1_∥_*∞*_≤*c*_1_.

Now, we will deduct a local growth bound on *Π* which will be utilized to prove Lemma 5.

From the definition of *f*, for fixed *y*, *Π* is a quadratic program with linear constraints. It is well known from optimization theory that this problem has a unique solution, see e.g. (Nash and Sofer 1996)[Theorem 14.4]. We can now apply Danskin’s theorem, see e.g. (Bonnans and Shapiro 2000)[Theorem 4.13], to conclude that *Π* is continously differentiable and $\nabla _{y}\Pi =\gamma f_{y}^{\prime }\left (\pi ^{\ast }\right) \in \mathbb {R}^{n}$. Calculations give now that ∇_*y*_*Π* is bounded ∀(*t*,*y*) on $[0,T]\times \mathbb {R}_{+}^{k}$. We are now equipped to prove Lemma 5 and 6.

#### **Proof of Lemma7**.

For brevity here ${Y_{u}^{y}}$ denotes *Y*_*u*_ with *Y*_0_=*y*. If the derivative of the integrand in equation (16) is absolutely integrable then by dominated convergence we may shift the order of derivative and expectations, see (Folland 1999)[Theorem 2.27]. Further,

$$\begin{aligned} &\mathbb{E}\left[ \left\vert \frac{\partial}{\partial y_{j}}e^{\omega_{T-t}(Y)}\right\vert |Y_{0}=y\right]\\ &=\mathbb{E}\left[e^{\omega_{T-t} (Y)}\left\vert \frac{\partial}{\partial y_{j}}\int_{0}^{T-t}\Pi\left(Y_{u}^{y}\right)du\right\vert |Y_{0}=y\right]\\ &\leq\mathbb{E}\left[e^{\omega_{T-t}(Y)}\int_{0}^{T-t}|\frac{\partial}{\partial y_{j}}\Pi \left({Y_{u}^{y}}\right)|du|Y_{0}=y\right]\\ &\leq(T-t)\sup_{u\in\left[ 0,T\right] }\left\vert \frac{\partial}{\partial y_{j}}\Pi\left({Y_{u}^{y}}\right)\right\vert \mathbb{E}\left[e^{\omega_{T-t}(Y)}|Y_{0} =y\right] \end{aligned} $$

which is finite by Lemma 4 and the finiteness of ∇_*y*_*Π*. Hence

$$\frac{\partial}{\partial y_{j}}h(t,y)=\mathbb{E}\left[\frac{\partial}{\partial y_{j}}e^{\omega_{T-t}(Y)}|Y_{0}=y\right]\quad,j=1,...,k. $$

#### **Proof of Lemma8**.

The first part follows from 5 by equation (19). Note that $|\frac {\partial }{\partial y_{j}}e^{\omega _{T-t}(Y^{y})}|$ is continuous in *t* and *y* so by (Folland 1999) [Theorem 2.27(a)] then $\frac {\partial }{\partial y_{j} }h(t,y)$ is continous in $(t,y)\in [0,T]\times \mathbb {R}_{+}^{k}$.

Thus, the lemmas 3, 4, 5 and 6 are proved, and by equation (17), $h_{y}^{\prime }$ is continuous. Hence, $h(t,y)\in C^{1,\mathbf {1}^{\mathcal {T}}}\left ([0,T]\times \mathbb {R}_{+}^{k}\right)$, as given in equation (16), is a well defined solution to the reduced HJB equation.

## Verification of the probabilistic representation of the solution

In Section 6,

$$v(t,x,y)=-e^{-\gamma x}h\left(t,y\right) =-e^{-\gamma x}\mathbb{E} \left[e^{\omega_{T-t}(Y)}\mid Y_{0}=y\right] $$

is conjectured to be a solution to the HJB equation. We have also that *v* is a solution to the actual optimization problem if all conditions in the Verification Theorem, theorem 1 hold. The three conditions are the following: suitable differentiability i.e. $v(t,x,y)\in C^{1,2,\mathbf {1}^{T}}\left ([0,T]\times (0,\infty)^{k+1}\right)$; boundedness of the quadratic variation of the Itō integrals i.e. equation (12); boundedness of the Levy measures, i.e. equation (13).

First, the diffentiablility part is trivial since by Section 6, $h(t,y)\in C^{1,\mathbf {1}}\left ([0,T]\times \mathbb {R}_{+}^{k}\right)$. Further, *v*(*t*,*x*,*y*) is infinitely differentiable in *x* and the desired result follows. Secondly,

$$\begin{aligned} \mathbb{E}\left[v_{x}\left(t,X_{t},Y_{t-}\right)^{2}\pi^{\mathcal{T}}\sigma_{t}\sigma _{t}^{\mathcal{T}}\pi\right] &\leq\mathbb{E}\left[v_{x}(t,X_{t},Y_{t-})^{2q}\right]^{\frac{1}{q}}\\ &\quad\times\mathbb{E} \left[\left(\pi^{\mathcal{T}}\sigma_{t}\sigma_{t}^{\mathcal{T}}\pi\right)^{p}\right]^{\frac{1}{p}} \end{aligned} $$

due to Hölder’s inequality with *p*,*q* conjugate exponents. The latter term is finite for every *p*∈[1,*∞*) by Lemma 2 since ∥*π*∥_*∞*_≤*c*_*π*_. Further, since *v*_*x*_(*t*,*x*,*y*)=−*γv*(*t*,*x*,*y*), another application of Hölder’s inequality yields that equation (12) is finite if

$$  {\int_{0}^{T}}\mathbb{E}\left[v(u,X_{u},Y_{u})^{2q}\right]du\,=\,{\int_{0}^{T}}\mathbb{E}\left[ e^{2q\left(\omega_{T-u}(Y)-\gamma X_{u}\right) }\right] du<\infty.   $$

We fix some *ε*>0 and let *q*=1+*ε*. Hölder’s inequality gives

(27) $$  \begin{aligned} {\int_{0}^{T}}\mathbb{E}\left[ e^{2\left(1+\epsilon\right) \left(\omega_{T-u}(Y)-\gamma X_{u}\right) }\right] du& \leq{\int_{0}^{T}}\mathbb{E}\left[e^{-2\hat{p}(1+\epsilon)\gamma X_{u}}\right]^{\frac {1}{\hat{p}}}\\ &\quad\times\mathbb{E}\left[e^{2\hat{q}(1+\epsilon)\omega_{T-t}(Y)}\right]^{\frac {1}{\hat{q}}}du, \end{aligned}  $$

where $\hat {p},\hat {q}$ are new conjugate exponents. Further, choose $\hat {p}=2/(1+\epsilon)$. We can now reason completely analogously to the deduction of Proposition 1 to see that the first part of the integrand is finite if

$$c_{2}\geq(4c_{\pi}\gamma\vee1)^{2}\left(2||\mu^{1}||_{\infty}+1\right). $$

In complete analogue to the deduction of Lemma 4, we have, since $\hat {q}=2/\left (1-\epsilon \right) $, that the second part of the integrand is finite if $8{\frac {1+\epsilon }{1-\epsilon }}c_{\pi }\left \Vert \mu ^{1}\right \Vert _{\infty }\leq c_{1}$. This gives the desired result.

Finally, by the definition of *v*(*t*,*x*,*y*) and Hölder’s inequality with *p*=4, then

$$\begin{aligned} &\mathbb{E}\left[\left\vert v(u,X_{u},Y_{u-}+{ze}_{i})-v(u,X_{u},Y_{u-})\right\vert \right]\\ &=\mathbb{E}\left[\left\vert e^{-\gamma X_{u}}\left(h(u,Y_{u-}+{ze}_{i})-h(u,Y_{u-})\right) \right\vert \right]\\ &\leq\mathbb{E}\left[ e^{-4\gamma X_{u}}\right]^{\frac{1}{4}} \mathbb{E}\left[ \left\vert h(u,Y_{u-}+{ze}_{i})-h(u,Y_{u-})\right\vert ^{\frac{4}{3}}\right]^{\frac{3}{4}}, \end{aligned} $$

for all *i*=1,…,*k*. The first term is identical to the first integrand term in equation (27), and hence finite. We see that the integral with respect to Lévy measure of the second term is finite by arguing completely analogously to the deduction of Lemma 4, but with $c_{L}\geq \frac {4}{3}{Tc}_{\Pi ^{\prime }}$. The desired result follows.

To conclude, the proposed function *v*(*t*,*x*,*y*) satisfies all conditions of Theorem 1, and thus the optimal value function *v*(*t*,*x*,*y*) is the solution to our optimization problem. Furthermore *π*^∗^, given by equation (24), is the optimal portfolio strategy.

### Remark 1.

Note that the portfolio optimization part of $\mathcal {H}h$, $\sup _{\pi \in \Gamma }\left \{\pi ^{T}\mu -\frac {\gamma }{2}\pi ^{T}\sigma \sigma ^{T}\pi \right \}$ completely replicates the problem of maximizing the investors expected wealth without exceeding a predetermined risk level in a one-period set up. Thus any optimal solution of our problem will correspond to consecutive solutions of the classical Markowitz mean-variance problem in continuum.

### Remark 2.

We can choose the bounds on our portfolio weights *π* such that the optimal strategy does not cause any of the investor’s total portfolio holdings, i.e. the portfolio weights *π* plus the benchmark weights, to become negative. This ensures that the investor’s wealth remains positive.
